# Genetic association and differential expression of *PITX2* with acute appendicitis

**DOI:** 10.1007/s00439-018-1956-2

**Published:** 2018-11-03

**Authors:** Ekaterina Orlova, Andrew Yeh, Min Shi, Brian Firek, Sarangarajan Ranganathan, 23andMe Research Team, David C. Whitcomb, David N. Finegold, Robert E. Ferrell, M. Michael Barmada, Mary L. Marazita, David A. Hinds, John R. Shaffer, Michael J. Morowitz

**Affiliations:** 1 Department of Human Genetics, Graduate School of Public Health, University of Pittsburgh, 130 De Soto Street, 3131 Parran Hall, Pittsburgh, PA 15261 USA; 2 Center for Craniofacial and Dental Genetics, University of Pittsburgh, Pittsburgh, PA 15219 USA; 3 Department of Oral Biology, School of Dental Medicine, University of Pittsburgh, Pittsburgh, PA 15261 USA; 4 Department of Surgery, University of Pittsburgh School of Medicine, Pittsburgh, PA 15261 USA; 5 Department of Pathology, University of Pittsburgh School of Medicine, Pittsburgh, PA 15261 USA; 6 Department of Division of Gastroenterology, Hepatology and Nutrition, University of Pittsburgh School of Medicine, Pittsburgh, PA 15261 USA; 7 Department of Cell Biology and Molecular Physiology, University of Pittsburgh, Pittsburgh, PA 15261 USA; 8 23andMe, Inc., Mountain View, CA 94041 USA; 9 Faculty Pavilion 7th Floor, Children’s Hospital of Pittsburgh of UPMC, 4401 Penn Avenue, Pittsburgh, PA 15224 USA

## Abstract

Appendicitis affects 9% of Americans and is the most common diagnosis requiring hospitalization of both children and adults. We performed a genome-wide association study of self-reported appendectomy with 18,773 affected adults and 114,907 unaffected adults of European American ancestry. A significant association with appendectomy was observed at 4q25 near the gene *PITX2* (rs2129979, *p* value = 8.82 × 10^−14^) and was replicated in an independent sample of Caucasians (59 affected, 607 unaffected; *p* value = 0.005). Meta-analysis of the associated variant across our two cohorts and cohorts from Iceland and the Netherlands (in which this association had previously been reported) showed strong cumulative evidence of association (OR = 1.12; 95% CI 1.09–1.14; *p* value = 1.81 × 10^−23^) and some evidence for effect heterogeneity (*p* value = 0.03). Eight other loci were identified at suggestive significance in the discovery GWAS. Associations were followed up by measuring gene expression across resected appendices with varying levels of inflammation (*N* = 75). We measured expression of 27 genes based on physical proximity to the GWAS signals, evidence of being targeted by eQTLs near the signals according to RegulomeDB (score = 1), or both. Four of the 27 genes (including *PITX2*) showed significant evidence (*p* values < 0.0033) of differential expression across categories of appendix inflammation. An additional ten genes showed nominal evidence (*p* value < 0.05) of differential expression, which, together with the significant genes, is more than expected by chance (*p* value = 6.6 × 10^−12^). *PITX2* impacts morphological development of intestinal tissue, promotes an anti-oxidant response, and its expression correlates with levels of intestinal bacteria and colonic inflammation. Further studies of the role of *PITX2* in appendicitis are warranted.

**Electronic supplementary material:**

The online version of this article (10.1007/s00439-018-1956-2) contains supplementary material, which is available to authorized users.

## Introduction

The appendix is commonly thought to be vestigial in humans (Cakmak et al. 2014), although recent reports have indicated a putative role for the appendix in maintaining a healthy gut microbiota (Donaldson et al. 2015). Regardless, inflammation of the appendix remains a major source of morbidity worldwide, particularly in developed countries. Acute appendicitis affects approximately 9% of Americans (Anderson et al. 2012) and is one of the most common reasons for emergent abdominal surgery. It is more common in males than females (1.4:1) and occurs most commonly in the second to third decades of life (Anderson et al. 2012; Körner et al. 1997). Roughly 30% of affected patients present with advanced disease featuring an appendiceal perforation, resulting in prolonged hospitalization and higher rates of complications (Barrett et al. 2013).

Historically, appendiceal obstruction from fecaliths or lymphoid hyperplasia has been considered the underlying cause of appendicitis (Holcomb and Murphy 2010). However, efforts to verify this theory experimentally have generally been unsuccessful, and pathologic reviews of appendectomy specimens have clearly shown that many or most cases of appendicitis occur in the absence of an obvious luminal obstruction (Carr 2000; Singh and Mariadason 2013; Chandrasegaram et al. 2012). For this reason, others have proposed that appendicitis, like other inflammatory processes, results from both genetic and environmental risk factors (Arnbjörnsson and Bengmark 1984; Ergul 2007; Sadr Azodi et al. 2009). For example, much evidence supports a role of lack of dietary fiber in the increased incidence of disease in developed nations (Adamidis et al. 2000; Arnbjörnsson 1983). Newer evidence has linked appendicitis with specific disturbances of the appendiceal microbiome (Zhong et al. 2014; Swidsinski et al. 2011, 2012), and this idea is supported by the fact that nonoperative therapy with antibiotics can successfully treat some patients with the disease (Minneci et al. 2016). Overall, there is no consensus on the pathophysiology underlying appendicitis, which appears to represent a unique disease process distinct from inflammatory disorders elsewhere in the gastrointestinal tract (Murphy et al. 2008).

The role of host genetics in predisposition to appendicitis is poorly understood, but the available evidence suggests that genetic factors contribute to susceptibility. For example, heritability estimates of appendicitis derived from linkage, complex segregation, and twin studies range between 27 and 56% (Basta et al. 1990; Duffy et al. 1990; Oldmeadow et al. 2009). Recently, association was observed for a locus on 4q25 near *PITX2* with appendicitis in Northern European adults (Kristjansson et al. 2017). This association was not found in children, suggesting potentially different genetic mechanisms or effect sizes of genetic risk factors for appendicitis between children and adults. Other genetic variants that account for the heritability of appendicitis have yet to be discovered.

Here, we report results of a genome-wide association study (GWAS) of appendectomy with the largest number of cases to date with independent replication. We follow up the association studies with an analysis of gene expression. Our results support the role of the 4q25 locus and *PITX2* in risk for appendicitis, and we nominate additional risk genes for the follow-up study.

## Methods

### GWAS

Research participants were from the personal genetics company 23andMe, Inc., and provided informed consent and participated in the research online, under a protocol approved by the external AAHRPP-accredited IRB, Ethical and Independent Review Services (E&I Review). DNA samples were provided via saliva collection kits and genotyped using one of four Illumina® (San Diego, USA) genotyping platforms (HumanHap550 BeadChip, OmniExpress + BeadChip, or one of two custom panels designed, in part, for comparability to these). Genetic data were imputed to the 1000 Genomes phase 1 reference using Minimac (Howie et al. 2012) and phased using Beagle (Browning and Browning 2007) (v 3.3.1) separately for the four genotyping platforms. SNPs were filtered for those with call rate > 90%, Hardy–Weinberg equilibrium *p* value > 10^−20^, MAF > 0.1%, and without evidence of a batch effect, or large allele frequency discrepancies compared to European 1000 Genomes reference data. SNPs were also flagged if present solely on the 23andMe V1 platform (due to small sample size), date effects (*p* value < 10^−50^), and if logistic regression results that did not converge due to complete separation. Imputed SNPs are represented by estimated allele dosage over a set of possible imputed genotypes and were included in the GWAS given satisfaction of quality metrics used in Minimac. Imputation quality and batch effects were evaluated using the average (avg.rsqr) and minimum (min.rsqr) of Minimac’s rsqr statistic aggregated over a series of imputation batches and an ANOVA test for a batch effect (p.batch) across these imputation batches (criteria: joint avg.rsqr > 0.5, min.rsqr > 0.3, and p.batch > 1 × 10^−50^).

Genetic association analyses were limited to unrelated participants with ≥ 97% European ancestry, as compared to HapMap2 populations (Falush et al. 2003), and were conducted using self-reported appendectomy as the phenotype in a cohort of 18,773 affected and 114,907 unaffected participants as described (Chang et al. 2015; Ferreira et al. 2014; Apfel et al. 2012). Those affected answered “yes” to “Have you ever had your appendix removed?” (answer choices: “yes,” “no,” and “I’m not sure”), and/or “Have you ever had any of the following other surgeries?” (answer choices to the “appendectomy” selection: “yes,” “no,” and “I don’t know.”) Unaffected individuals answered “no,” and those who responded with discordant results to the two questions or answered “I don’t know/I’m not sure” were excluded from the study.

Tests of genetic association were performed using logistic regression while assuming an additive genetic model and adjusting for age, sex, and the top five principal components of ancestry. The genomic control procedure was used to account for variance inflation not effectively controlled for by principal components; the results were adjusted for a genomic inflation factor of 1.034 (Devlin and Roeder 1999). The thresholds for genome-wide significance and suggestive significance were set at *p* values = 5 × 10^−8^ and 1 × 10^−6^, respectively.

### Replication

The top SNPs for seven of the nine loci reaching genome-wide or suggestive significance (*p* value < 1 × 10^−6^) in the GWAS were tested for genetic association in an independent replication cohort of non-Hispanic European ancestry. Two of the nine SNPs could not be tested in the replication cohort due to low MAF, and no surrogate SNPs in high LD (*r*^2^ > 0.8) were available. The replication cohort was sourced from the Center for Oral Health Research in Appalachia cohort 1 (COHRA1), a study of oral health in a rural population described previously (Polk et al. 2008). COHRA1 included data collection on prior hospitalizations and operations as part of a medical history survey. Appendicitis cases were ascertained based on self-report of appendicitis or appendectomy. 59 appendicitis cases of any age and 607 unaffected adults over age 30 were included in the replication analyses. A minimum age of 30 was chosen for defining unaffected participants to reduce the chances of including individuals who were susceptible, but had not yet had appendicitis (Körner et al. 1997). Logistic regression was performed assuming an additive model and adjusting for sex and the first principal component of ancestry. In light of multiple comparisons, the significance threshold to declare replication was determined by Bonferroni adjustment to be *p* value = 0.007.

### Meta-analysis

A fixed effects meta-analysis was performed for the top SNP (rs2129979) across four cohorts and heterogeneity was tested using Cochrane’s *Q* statistic in Plink v1.9 (Purcell et al. 2007). Meta-analysis results were visualized in a forest plot created in R (v3.4.2). The analysis included results from the 23andMe and COHRA1 cohorts, as well as two cohorts of Icelandic and Dutch ancestry described previously (Kristjansson et al. 2017). The Icelandic cohort consisted of 7267 affected individuals ascertained through medical record review and 327,134 unaffected people recruited for genetics research through deCODE. The Dutch cohort included 1139 affected people based on self-report of appendicitis/appendectomy and 4587 unaffected people, with both groups drawn from several cancer and other research studies.

### Appendiceal gene expression

75 appendix samples were collected from children (aged 5–18 years; mean = 10.9 years) undergoing appendectomy at Children’s Hospital of Pittsburgh of UPMC following parental informed consent under approved IRB protocol (University of Pittsburgh #PRO14090296). The samples were classified based on histopathology as mildly inflamed (*N* = 8), severely inflamed (*N* = 38), perforated (*N* = 18), or uninflamed (*N* = 11; due to incidental appendectomy) and were preserved as formalin-fixed paraffin-embedded tissue. A custom panel of 27 genes was selected for measurement of gene expression based on multiple lines of evidence. Genes were prioritized based on proximity to the associated variants observed in the GWAS, with preference given to genes with biological plausibility and those nearest to the most strongly associated variants. Genes were also prioritized based on greatest evidence of regulation by expression quantitative trait loci (eQTLs) near the GWAS signals, which was defined as a RegulomeDB score of 1, “likely to affect binding and linked to expression of a gene target” (Table S1). Genes targeted by eQTLs across multiple associated loci were prioritized. eQTL locations and target genes were obtained from RegulomeDB (version 1.1, publicly available at regulome.stanford.edu) (Boyle et al. 2012).

NanoString Technologies’ nCounter™ Gene Expression Assay was used to measure gene expression (NanoString Technologies; Seattle, WA). RNA was extracted from FFPE appendix tissue samples with the PureLink FFPE Total RNA Isolation Kit (ThermoFisher®). 100 ng of RNA was used as input for the protocol recommended by the manufacturer. RNA extraction and nCounter assay were performed at the University of Pittsburgh HSCRF Genomics Research Core. Results from the Nanostring were normalized using the nSolver analysis software 2.5 (NanoString). Samples underwent quality filtering using default parameters. Six positive and eight negative spiked-in controls were used. Gene expression levels were normalized to those of eight housekeeping genes listed in Table S1. Genes were tested for differential expression across categories of appendix sample inflammation or perforation using the nonparametric Cruzick trend test. A *p* value threshold of 0.0033 for declaring statistical significance given the effective number of independent tests was determined using the Li and Ji method (2005). The Kruskal–Wallis test was done as a secondary analysis to check for differences that were not monotonic across severity categories. The Wilcoxon rank sum test was used to compare inflamed or perforated and uninflamed tissue only for genes surpassing nominal statistical significance (*p* value < 0.05) via the Kruskal–Wallis test. Enrichment of nominally or significantly differential expression across genes in the expression panel was determined using the binomial test.

## Results

**Fig. 1:**
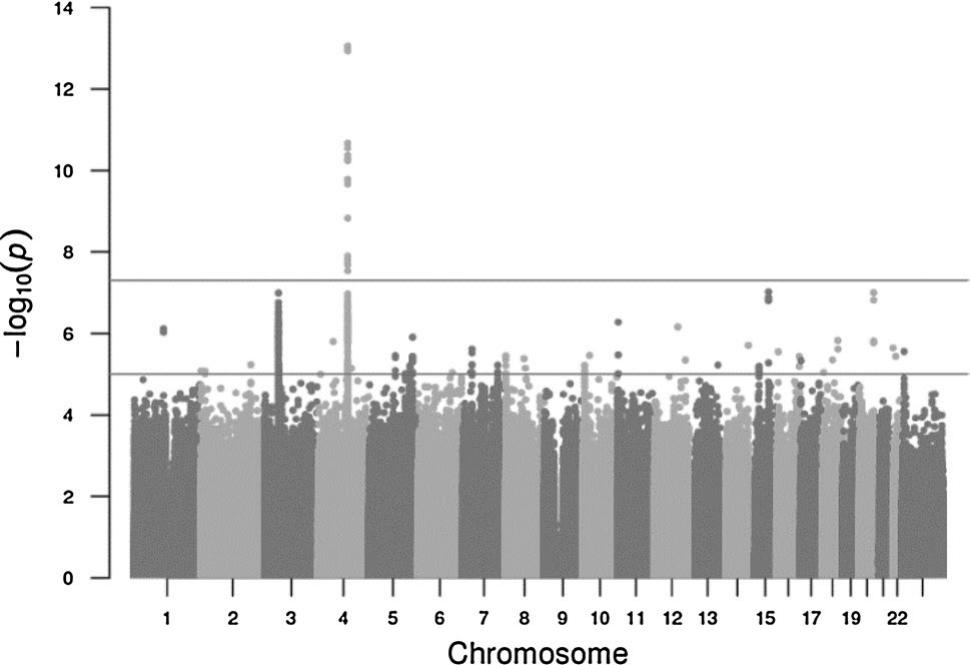
Manhattan plot of GWAS of self-report of appendectomy in the 23andMe (discovery) cohort. The upper horizontal line represents the threshold for genome-wide significance (*p* value < 5 × 10^−8^), and the lower horizontal line represents suggestive significance (*p* value < 1 × 10^−6^)

**Fig. 2:**
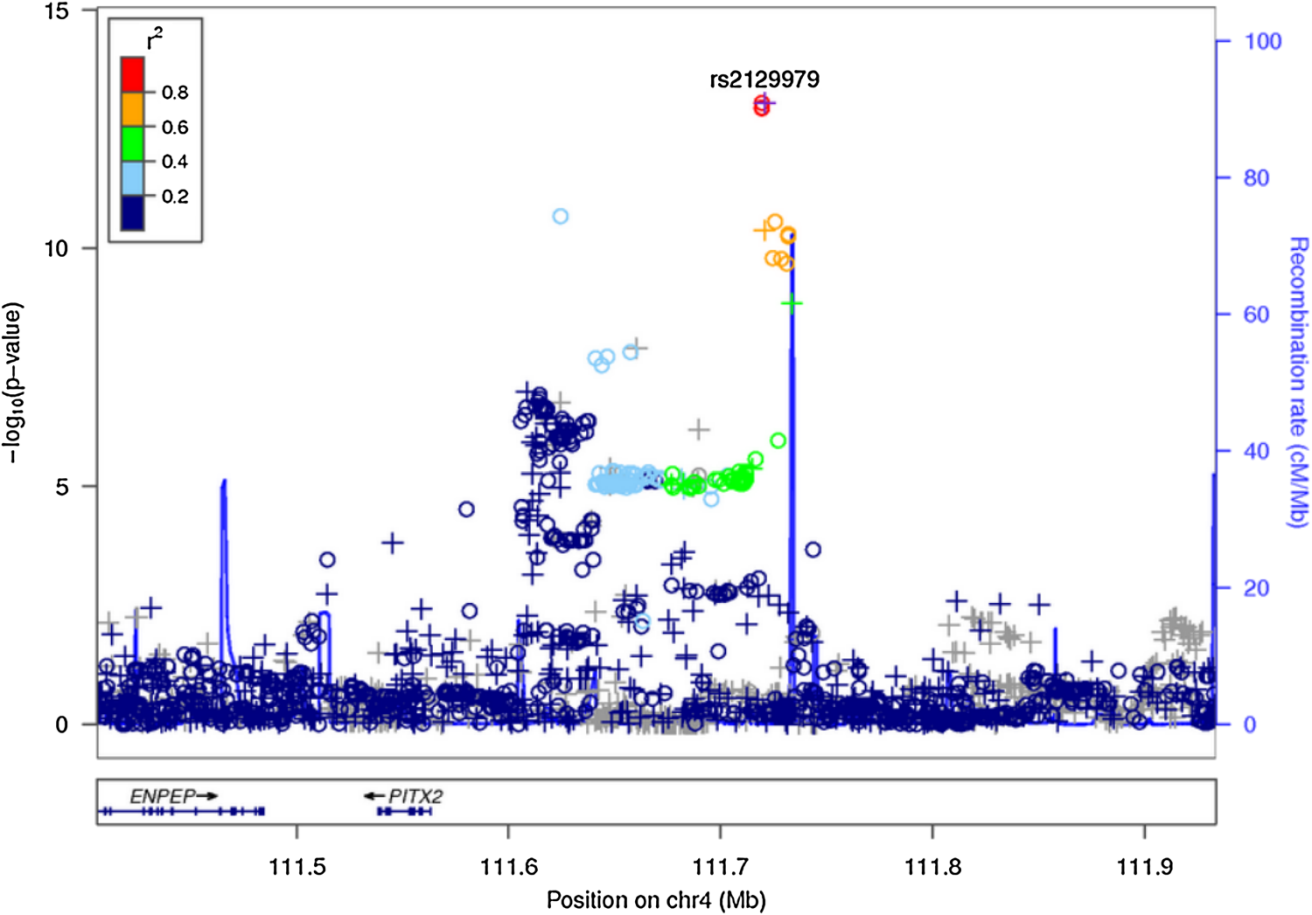
Regional association plot of the genome-wide significant rs2129979 in the 23andMe GWAS. Blue lines indicate the recombination rate plotted along the right Y-axis. The colors indicate the strength of linkage disequilibrium (LD) with the index SNP. Genes near the lead SNP are shown along the X-axis. The symbol “+” indicates a genotyped SNP; open circle indicates an imputed SNP

A GWAS of appendectomy was conducted on 133,680 individuals. Participant demographics are shown in Table 1. Results of the GWAS are shown in the Manhattan plot in Fig. 1. No evidence of genomic inflation was detected (genomic inflation factor, *λ*, was 1.034; see quantile–quantile plot in Figure S1). One genome-wide significant locus was observed on chromosome 4q25 near *PITX2* (lead SNP rs2129979, *p* value = 8.82 × 10^−14^, OR = 1.10, 95% CI 1.07–1.13; Fig. 2; Table 2). Genetic association with similar effect size has recently been reported at this locus in a GWAS of Northern European adults (OR = 1.15, 95% CI 1.10–1.20) (Kristjansson et al. 2017). Eight other loci reached suggestive significance (*p* value < 1 × 10^−6^; Table 2), including 15q24 (*NEO1*), 20q13 (*RBM38, CTCFL*), 3p21 (*TRAIP*), 4q25 (*c4orf32*), 11p15 (*AP2A2*), 12q21 (*DUSP6*), 1p13 (*CD53*), and 3p21 (*RAD54L2*) (Figure S2).

**Table 1:** Cohort demographics of the 23andMe (discovery) and COHRA1 (replication) data sets

	23andMe		COHRA1	
	Affected	Unaffected	Affected	Unaffected
Total, *N* (%)	18,773 (14%)	114,907 (86%)	59 (9%)	607 (91%)
Female (%)	56.5	47.9	52.5	59.8
Male (%)	43.5	52.1	47.5	40.2
Age (years) (%)				
(0–30)	4.1	13	30.5	0
(30–45)	14.4	28.8	55.9	78.7
(45–60)	25.7	27.6	13.6	19.6
(60+)	55.9	30.5	0	1.6

The ages represent ages at which the research participants were genotyped

**Table 2:** Replication results: summary statistics for loci reaching suggestive significance (*p* value < 1 × 10^−6^) in the 23andMe cohort, and their corresponding statistics in the COHRA1 replication cohort

Cyto	SNP	BP	Effect allele^b^	MAF	Discovery (23andMe)			Replication (COHRA1)^a^		
					OR	95% CI	*p* value^c^	OR	95% CI	*p* value^c^
4q25	rs2129979	111720997	G	0.31	1.101	1.073, 1.129	$8.82 \times 1{0^{ - 14}}$	1.78	1.194, 2.639	**0.0046**
15q24	rs192656182	7359997	T	0.015	1.453	1.272, 1.660	9.5 × 10^−8^			
20q13	rs137882920	5600525	T	0.019	0.745	0.666, 0.833	9.91 × 10^−8^			
3p21	rs2247036	4988234	C	0.47	0.939	0.917, 0.962	1.01 × 10^−7^	0.73	0.494, 1.071	0.1068
4q25	rs17044095	11277741	G	0.24	0.931	0.906, 0.957	3.23 × 10^−7^	1.04	0.654, 1.667	0.8569
11p15	rs117367662	96782	T	0.05	0.863	0.814, 0.915	5.29 × 10^−7^	1.40	0.578, 3.366	0.4590
12q21	rs1650337	8977006	T	0.001	Inf	1.099, Inf	6.95 × 10^−7^			
1p13	rs75972139	111373721	A	0.01	1.250	1.147, 1.364	7.77 × 10^−7^	2.65	0.849, 8.295	0.0933
3p21	rs6445791	5160198	G	0.12	1.084	1.050, 1.120	9.62 × 10^−7^	1.08	0.650, 1.804	0.7588

*MAF* Minor allele frequency in Europeans, *Cyto* SNP cytogenetic location, *Inf* infinity

^a^Some 23andMe lead SNPs were unavailable in COHRA1 due to low MAF, thus have blank values in replication

^b^The effect allele is the minor allele

^c^Significantly associated SNPs in 23andMe (*p* value < 5 × 10^−8^) and COHRA1 (*p* value < 0.05) are bold

The lead SNPs of seven of the nine loci identified in the GWAS were considered for replication testing in 666 individuals from the COHRA1 cohort. Note, neither lead SNPs nor surrogates in high LD were available for three of the nine loci due to low MAF. COHRA1 cohort demographics are listed in Table 1. Evidence of replication was observed for rs2129979 (*p* value = 0.0046), the lead SNP at the significant locus on 4q25 near *PITX2*, after adjustment for multiple testing. The direction of effect of the SNP in the replication sample (OR = 1.78, 95% CI 1.194–2.639) was the same as in the 23andMe sample. No other SNPs showed evidence of replication. Replication results are detailed in Table 2.

**Fig. 3:**
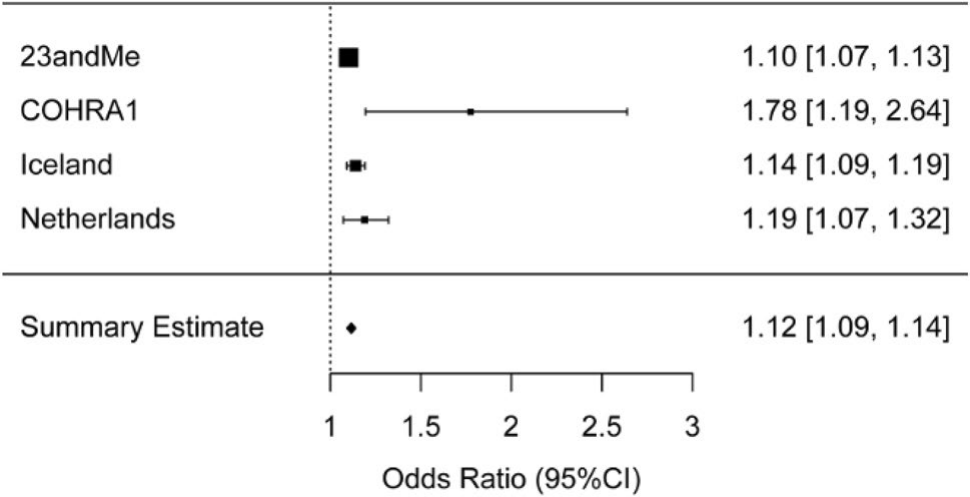
Forest plot for rs2129979. Odds ratios for appendicitis/appendectomy are shown as squares proportional to the sample size. Error bars indicate the 95% confidence interval. The fixed effects meta-analysis odds ratio estimate and confidence interval are represented by the position and width of the diamond

A meta-analysis was conducted on the most significantly associated SNP (rs2129979) in the 23andMe GWAS across the 23andMe, COHRA1, Icelandic (OR = 1.14; 95% CI 1.09–1.19; *p* value = 3.5 × 10^−9^), and Dutch cohorts (OR = 1.19; 95% CI 1.07–1.32; *p* value = 0.0011) (Fig. 3). The direction of effect was consistent for all four groups. Statistically significant genetic heterogeneity at this locus was found using Cochran’s *Q* statistic (*p* value = 0.0336), indicating some between-cohort differences in the genetic effects of this locus.

**Fig. 4:**
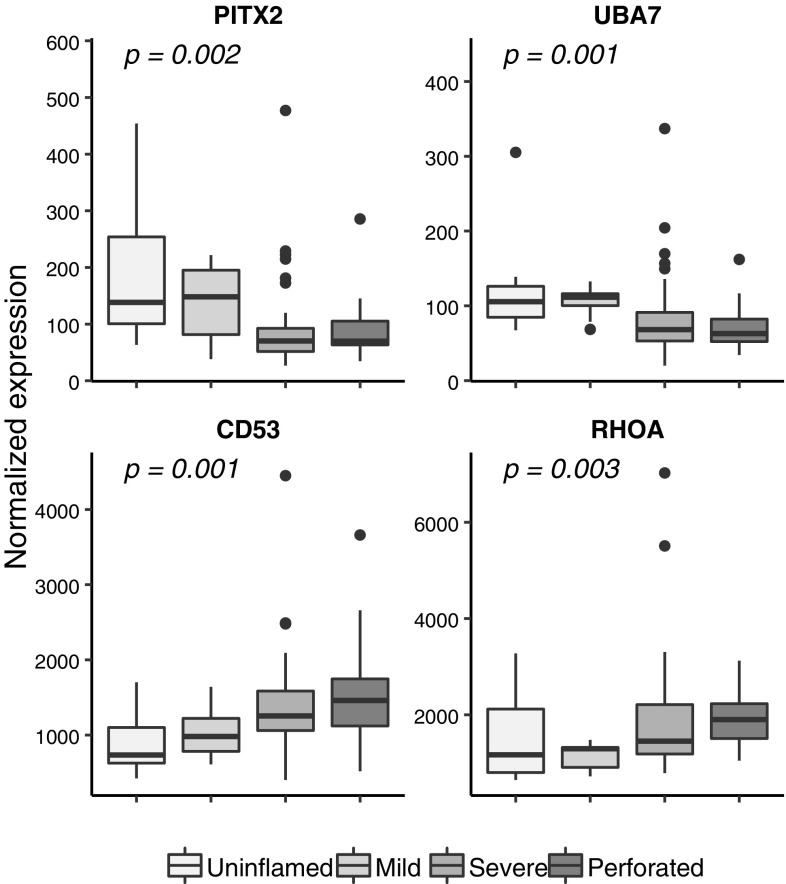
Genes showing differential expression across uninflamed, mildly inflamed, severely inflamed, and perforated pediatric appendix samples by the trend test. The Y-axis depicts the normalized count of the number of transcripts for the genes. Horizontal lines indicate the median expression values, boxes represent the interquartile range, whiskers extend to the most extreme point within 1.5 × interquartile range of the box, and points represent extreme values falling outside the whiskers

Consistent with most genetic associations for complex diseases, the associated SNPs near *PITX2* and other suggestive loci observed in this GWAS are non-coding, and statistical evidence of association at these loci does not point to specific mechanisms through which they impact susceptibility to appendicitis. Non-coding variants, such as these, may regulate gene expression. Therefore, we investigated expression levels for a panel of candidates prioritized based on the GWAS results. Expression levels in a custom panel of 27 genes were measured in 75 appendix samples (11 uninflamed, 8 mildly inflamed, 38 severely inflamed, 18 perforated) by direct detection of RNA barcodes. The mean age of the children from whom the samples were collected was 10.9 (range 5–18 years). Of the 27 genes tested, four genes showed significant differential expression across categories of appendix inflammation or perforation using a nonparametric trend test: *PITX2* (*p* value = 0.002), *UBA7* (*p* value = 0.001), *CD53* (*p* value = 0.001), and *RHOA* (*p* value = 0.003) (Fig. 4). Additionally, ten genes showed nominally significant (*p* value < 0.05) evidence of differential expression (*ENPEP p* value = 0.006; *NEO1 p* value = 0.023; *GMPPB p* value = 0.010; *MST1 p* value = 0.019; *MON1A p* value = 0.011; *FAM212A p* value = 0.006, *AP2A2 p* value = 0.019, *KCNA3 p* value = 0.036, *RAD54L2 p* value = 0.024, *WDR6 p* value = 0.013) (Figure S3). Moreover, the expression panel, overall, showed significant (*p* value < 6.6 × 10^−12^) enrichment of nominally or significantly differentially expressed genes (14 of 27), compared to what would be expected for this panel of genes by chance if there truly were no expression differences across the inflammation groups. Analyses using the Kruskal–Wallis test showed similar results to the trend test (Figures S4 and S5) and did not yield additional significant findings. *PITX2*, the gene closest to the most significant replicated GWAS signal (rs2129979), was among the genes showing differential gene expression. eQTLs and corresponding GWAS signals for each gene on the expression panel, and statistical results of expression analysis are listed in Table S1.

## Discussion

### GWAS replicates PITX2 signal at 4q25

This GWAS of appendectomy in 18,773 affected and 114,907 unaffected individuals of European ancestry identified one genome-wide significant locus (lead SNP rs2129979 at 4q25), which was replicated in a second cohort. This is the same SNP recently reported in a GWAS of appendicitis in Dutch and Icelandic cohorts (Kristjansson et al. 2017). Meta-analysis of rs2129979 across these four groups showed strong cumulative evidence of association (OR = 1.12; 95% CI 1.09–1.14; *p* value = 1.81 × 10^−23^) and weaker evidence of heterogeneity in effect size (Cochran’s *Q p* value = 0.0336).

The gene nearest the association signal is *PITX2*, which encodes the transcription factor Paired-Like Homeodomain 2. PITX2 plays a role in tissue-specific cell proliferation and left–right asymmetry during fetal development, and is important in morphogenesis of the cecum, the location in the gut at which the appendix develops in the embryo (Ryan et al. 1998; Essner et al. 2000; Logan et al. 1998). *PITX2* is also associated with Mendelian organ morphogenesis disorders, such as Axenfeld-Rieger (Fitch and Kaback 1978) and others (Cheong et al. 2016; Mattos et al. 1980). The 4q25 locus is also associated with atrial fibrillation (Gudbjartsson et al. 2007), but the specific associated SNPs are distinct from those associated with appendicitis (Kristjansson et al. 2017). Over- and under-expression of *PITX2* is also associated with atrial fibrillation (Syeda et al. 2017; Chinchilla et al. 2011; Pérez-Hernández et al. 2016), and PITX2 represses translation of genes that encode transcriptional regulators, ion channels, and cell junction proteins in the heart (Tao et al. 2014).

### Differential gene expression across appendix inflammation categories

The significant and suggestive variants observed in the GWAS were not protein coding, suggesting possible regulatory functions. Genetic association results were followed by an investigation into whether genes in associated regions and/or those targeted by eQTLs in the regions showed differential expression across uninflamed, mildly inflamed, severely inflamed or perforated appendix tissue samples. With this approach, we identified significant trends in gene expression across inflammation categories for four of 27 genes (*PITX2, UBA7, CD53*, and *RHOA*), and nominally significant trends for 10 genes. Though the expression differences of these latter genes were not significant after multiple testing correction, the expression panel, overall, was enriched for nominally and significantly differentially expressed genes (*p* value = 6.6 × 10^−12^). This may be due to global differences in expression across inflammation categories, or because the selection procedure based on the GWAS results yielded a panel of genes with differential expression.

Previous expression studies of inflamed appendices identified inflammatory gene expression signatures specific to mediators of the innate immune response and distinct from inflammatory bowel diseases (Yoon et al. 2002; Paajanen et al. 2002; Mazzucchelli et al. 1994; Dalal et al. 2005; Zeillemaker et al. 1996; Murphy et al. 2008). Our gene expression panel focused on following up specific GWAS results and did not include any genes whose expression has been previously associated with appendicitis. The fold-change of the significant genes’ expression ranged up to approximately twofold across conditions; although this is not as dramatic as the expression changes in inflammation-related genes previously observed in appendicitis (Murphy et al. 2008), these small changes may be biologically meaningful. Indeed, a fold-change of 2.2 in *PITX2* expression produces laterality defects in a mouse model (Bentham et al. 2010). While trends in gene expression of candidates identified in the GWAS lend additional support for their possible role in genetic susceptibility, we did not explicitly test the effect of the genetic variants on gene expression, and these results do not indicate the mechanisms through which they impact disease risk.

### Potential roles of PITX2 in appendicitis

Among the risk factors for appendicitis are diet, variation in the intestinal microbiome, and genetics, which may interact in promoting inflammation. For example, a low-fiber diet can influence intestinal microbiome composition and health outcomes by allowing microbes to contact the intestinal epithelium, leading to inflammation (Arnbjörnsson 1983; Veronese et al. 2018; Desai et al. 2016). Appendiceal microbiota in particular are known to be affected by both dietary intake and genetics (Goodrich et al. 2014), and several studies have found evidence for a microbial role in appendicitis (Swidsinski et al. 2011, 2012; Zhong et al. 2014; Jackson et al. 2014).

Based on its associations with diet, intestinal inflammation, and the intestinal microbiome, we hypothesize that tissue-specific *PITX2* expression may interact with these factors to contribute to the development of appendicitis. Specifically, *PITX2* expression is diet-dependent (Bentham et al. 2010) and negatively associated with colonic inflammation (Suzuki et al. 2007), a trend also found in appendiceal inflammation in this study. Additionally, decreased *PITX2* expression correlates with increased abundance of *Enterobacteriaceae* (Steegenga et al. 2017), a class of intestinal microbes often seen at times of inflammation (Lupp et al. 2007).

Another hypothesis is that PITX2 affects appendicitis risk during development, given its role as an important regulator during intestinal development (Fitch and Kaback 1978; Nichol and Saijoh 2011). It is plausible that PITX2-related appendiceal anatomical differences predispose to appendicitis (Kristjansson et al. 2017).

Further, we speculate that PITX2 may be involved in anti-oxidant or regenerative responses in the appendix. In the heart PITX2 promotes regeneration after injury through anti-oxidant response (Tao et al. 2016), and in the eye it may mediate the response to oxidative stress (Paylakhi et al. 2011; Strungaru et al. 2011), where it targets a pro-inflammatory cytokine (Moazzeni et al. 2016) and a gene encoding response to oxidative stress (Strungaru et al. 2011). PITX2 also promotes skeletal muscle regeneration through its repression of miR-31 (Vallejo et al. 2018). Inhibition of miR-31 in the colon alleviates colonic inflammation (Shi et al. 2017). Thus if PITX2 affects miR-31 in the appendix, it could help explain its association with the inflammation in appendicitis; indeed, miR-31 is abnormally expressed in multiple inflammatory diseases (Shi et al. 2017). However it is unclear to what extent target genes of PITX2 in various organs are concordant with those in the appendix given the scant overlap of its targets in muscle, eye, and other tissues (Strungaru et al. 2011).

### Additional gene candidates for appendicitis

We nominate the gene candidate *RHOA* (Ras Homolog Family Member A), which is located near the GWAS signal at 3p21 (lead SNP rs2247036), targeted by by 22 eQTLs (Table S1) and shows a significant trend of increasing expression with increasing appendix inflammation. RHOA is involved in signal transduction, actin cytoskeleton dynamics, and its overexpression is associated with tumor growth (Chen et al. 2016). It is required for intestinal epithelial cell integrity (Chung et al. 2015), controls intestinal stem cell regeneration in development and after injury or inflammation(Liu et al. 2017), and is upregulated in response to endotoxin (Cetin et al. 2004). These functions suggest a multifactorial cause of appendicitis in which multiple pathways—including an inability to repair damage—converge to predispose to the disease.

Another notable nominally differentially expressed (*p* value = 0.009) candidate gene, *MST1*, encodes macrophage stimulating protein. It is targeted by the eQTL rs11718165 near the GWAS signal at 3p21 (lead SNP rs2247036), and is associated with susceptibility to inflammatory bowel diseases (Fisher et al. 2008). MST1 regulates the innate immune response (Wu et al. 2018) (Galan and Avruch 2016), and biallelic *MST1* loss causes an immunodeficiency syndrome (Galan and Avruch 2016).

## Conclusions and limitations

A limitation of the study is the sample size of the COHRA1 replication cohort, which was underpowered to detect the identified signals in the discovery dataset. Nevertheless, the lead discovery SNP showed evidence of replication, and additional evidence from the Icelandic/Dutch cohorts underscores the strength of evidence for this locus. In addition, it is possible that alternative sources of eQTL target data other than RegulomeDB (e.g., GTEx, which was inaccessible during study design) would have led to different choices of genes for the expression panel and led to additional insights.

In this study, the appendicitis phenotypes were collected by self-report in both 23andMe and COHRA1 cohorts. Despite the frequency of incidental appendectomies (i.e., those not due to appendicitis) in the population and limitations of personal recall of medical procedures, self-report of this trait produced a viable phenotype that yielded a strong association in this GWAS and was replicated in the independent cohort. Phenotype misclassification, if present, would bias the GWAS toward the null hypothesis of no association, but would not cause false positive results. Therefore, true associations may have gone unobserved, whereas the observed associations, if false, were not due to limitations in the phenotype data collection. The fact that the strong previously reported association at *PITX2* was observed in both of our cohorts supports the utility of self-reported appendicitis phenotypes.

Appendix samples from the expression panel were drawn from a pediatric population, and it is possible that samples from adults might have yielded different results. Our expression experiment was designed prior to the publication of Kristjansson’s appendicitis GWAS (Kristjansson et al. 2017), in which the lead GWAS SNP near *PITX2* showed stronger association with disease with increasing age of onset. We cannot determine whether the same pattern holds true in our discovery cohort given that 23andMe did not collect data on age at the time of appendectomy. Moreover, the COHRA1 cohort included too few affected participants to evaluate genetic association across age-of-onset strata. Given the potential differences in pathophysiology between pediatric and adult-onset appendicitis it is a strength that we do not have a mixed age group of appendices, however, our results can only be interpreted in the context of pediatric-onset disease and cannot be generalized to adult-onset disease.

Future work to further understand the role of *PITX2* and other gene candidates in appendicitis would be to investigate potential eQTL effects on expression data and correlate those with genotype data on the tissue samples. Gene expression analyses in adult populations may also elucidate differences and similarities in pediatric versus adult-onset appendicitis.

Understanding the genetic factors influencing appendicitis may help elucidate the etiology of the disease and ultimately may inform systems for classifying patients by disease risk for optimal management. Further studies to understand the genetic contributors may also lead to more accurate diagnosis, more targeted treatment, and ultimately personalized prevention of this common disease.

## Electronic supplementary material

Below is the link to the electronic supplementary material.

Supplementary material 1 (DOCX 2591 KB)
