# Supplementary material for: Genetic association and differential expression of PITX2 with acute appendicitis
Source: Hum Genet. 2018 Nov 3;138(1):37–47. doi: 10.1007/s00439-018-1956-2 (PMC6514078; doi:10.1007/s00439-018-1956-2)
Supplement: Supplementary file 1 — Supplementary material 1 (DOCX 2591 KB) [file 439_2018_1956_MOESM1_ESM.docx]

**Supplementary Information**

**Supplementary Table 1** Genes included on expression panel of resected appendices. Gene Characteristics shows evidence for inclusion of genes on panel. eQTL(s) Targeting Gene – rsIDs of eQTLs near GWAS signal targeting a given gene. Near GWAS signal – X specifies genes within +/-500 kb of a GWAS signal. NA = SNP not tested for replication. Pair-wise tests are shown for genes surpassing nominal significance (p-value<0.05) by the Kruskall-Wallis test. P-values surpassing nominal significance are bolded and those surpassing statistical significance are marked with **. * Housekeeping gene used for normalization of gene panel expression data.

| **Gene** | **Gene Characteristics** | | **Locus** | **23andMe GWAS**  **p-value** | **Replication**  **p-value** | **Trend Test**  **P-value (Cuzick)** | **Differential Expression P-value (Kruskall-Wallis)** | **Pair-wise Differential Expression P-value**  **(Wilcoxon rank sum)** | | |
| --- | --- | --- | --- | --- | --- | --- | --- | --- | --- | --- |
|  | **eQTL(s) Targeting Gene** | **Near GWAS signal** |  |  |  |  |  | **Uninflamed**  **- Mild** | **Uninflamed**  **- Severe** | **Uninflamed**  **- Perforated** |
| *PITX2* |  | **X** | 4q25 | 9.12x10^-14^ | 0.0046 | **0.002**** | **0.003** | 0.540 | **0.002** | **0.004** |
| *ENPEP* |  | **X** | 4q25 | 9.12x10^-14^ | 0.0046 | **0.006** | 0.051 |  |  |  |
| *NEO1* | rs9920504 | **X** | 15q24 | 9.82x10^-8^ | NA | **0.023** | **0.016** | 0.970 | **0.028** | 0.120 |
| *HCN4* |  | **X** | 15q24 | 9.82x10^-8^ | NA | 0.088 | 0.396 |  |  |  |
| *CTCFL* |  | **X** | 20q13 | 1.02x10^-7^ | NA | 0.817 | 0.988 |  |  |  |
| *GMPPB* | rs1060970 | **X** | 3p21 | 1.04x10^-7^ | 0.1068 | **0.010** | 0.066 |  |  |  |
| *TRAIP* |  | **X** | 3p21 | 1.04x10^-7^ | 0.1068 | 0.561 | 0.425 |  |  |  |
| *UBA7* | rs1060970 | **X** | 3p21 | 1.04x10^-7^ | 0.1068 | **0.001**** | **0.005** | 0.970 | **0.014** | **0.004** |
| *IP6K1* |  | **X** | 3p21 | 1.04x10^-7^ | 0.1068 | 0.299 | 0.301 |  |  |  |
| *APEH* | rs4955430, rs3448, rs2140270, rs13096474, rs940045, rs1464569, rs8897, rs9860055, rs13322887, rs12583, rs1982861, rs10865956, rs9862534, rs4241407, rs2312462, rs6446298 | **X** | 3p21 | 1.04x10^-7^ | 0.1068 | 0.262 | 0.294 |  |  |  |
| *CDHR4* |  | **X** | 3p21 | 1.04x10^-7^ | 0.1068 | 0.463 | 0.509 |  |  |  |
| *RNF123* | rs9863142, rs9818758, rs6446298 | **X** | 3p21 | 1.04x10^-7^ | 0.1068 | 0.537 | 0.680 |  |  |  |
| *MST1R* |  | **X** | 3p21 | 1.04x10^-7^ | 0.1068 | 0.070 | 0.272 |  |  |  |
| *MST1* | rs11718165 | **X** | 3p21 | 1.04x10^-7^ | 0.1068 | **0.019** | **0.009** | 0.540 | 0.060 | **0.045** |
| *CAMKV* |  | **X** | 3p21 | 1.04x10^-7^ | 0.1068 | 0.522 | 0.563 |  |  |  |
| *MON1A* | rs9813644, rs6795772 | **X** | 3p21 | 1.04x10^-7^ | 0.1068 | **0.011** | **0.040** | 0.260 | 0.110 | **0.026** |
| *FAM212A* |  | **X** | 3p21 | 1.04x10^-7^ | 0.1068 | **0.006** | **0.040** | 1.000 | 0.082 | **0.011** |
| *MUC6* |  | **X** | 11p15 | 1.04x10^-7^ | 0.4590 | 0.699 | 0.704 |  |  |  |
| *AP2A2* |  | **X** | 11p15 | 1.04x10^-7^ | 0.4590 | **0.019** | **0.042** | 0.710 | 0.420 | 0.062 |
| *MUC2* |  | **X** | 11p15 | 1.04x10^-7^ | 0.4590 | 0.158 | 0.557 |  |  |  |
| *CHID1* |  | **X** | 11p15 | 1.04x10^-7^ | 0.4590 | 0.278 | 0.146 |  |  |  |
| *CD53* |  | **X** | 1p13 | 1.04x10^-7^ | 0.0933 | **0.001**** | **0.011** | 0.200 | **0.016** | **0.0037** |
| *KCNA3* |  | **X** | 1p13 | 1.04x10^-7^ | 0.0933 | **0.036** | 0.205 |  |  |  |
| *VPRBP* |  | **X** | 3p21 | 1.04x10^-7^ | 0.1068 | 0.911 | 0.805 |  |  |  |
| *RAD54L2* |  | **X** | 3p21 | 1.04x10^-7^ | 0.1068 | **0.024** | 0.122 |  |  |  |
| *RHOA* | rs1491983, rs2029591, rs4855833, rs2131108, rs9883000, rs9824435, rs2329021, rs1078341, rs6774202, rs4855885, rs11130217, rs11709734, rs12715437, rs11720705, rs7628207, rs9849038, rs9853352, rs9829155, rs7637711, rs6809879, rs1996663, rs1996664 |  | 3p21 | 1.04x10^-7^ | 0.1068 | **0.003**** | **0.016** | 0.710 | 0.130 | 0.056 |
| *WDR6* | rs13078949, rs6795772, rs17650792, rs7621003, rs6797765, rs2177268, rs6784820, rs11715915, rs1464567, rs1464566, rs8897, rs885592, rs10865955, rs4855864, rs7637999, rs11130199, rs3870338, rs1050088, rs4241407, rs2312462, rs2029591, rs2329020, rs2131104, rs4855881, rs11130214, rs2291542, rs9853352 |  | 3p21 | 1.04x10^-7^ | 0.1068 | **0.013** | **0.006** | 0.170 | **0.038** | 0.069 |
| *UBE2D2** |  |  |  |  |  |  |  |  |  |  |
| *RNF20** |  |  |  |  |  |  |  |  |  |  |
| *CNOT2** |  |  |  |  |  |  |  |  |  |  |
| *HDAC3** |  |  |  |  |  |  |  |  |  |  |
| *EIF2C1** |  |  |  |  |  |  |  |  |  |  |
| *TOX4** |  |  |  |  |  |  |  |  |  |  |
| *GIGYF2** |  |  |  |  |  |  |  |  |  |  |
| *C2orf42** |  |  |  |  |  |  |  |  |  |  |


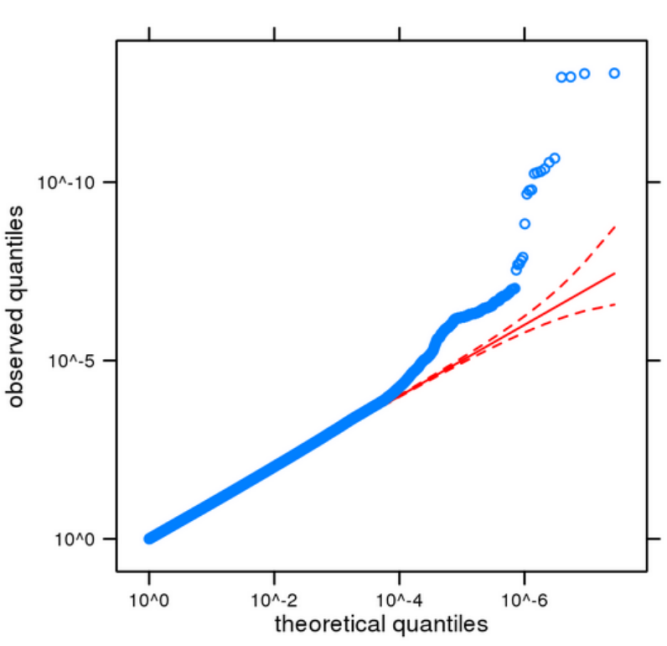


**Supplementary Fig 1** Quantile-quantile plot of the 23andMe Appendectomy GWAS. The genomic control inflation factor λ was 1.034. The dashed red lines represent the 95% confidence envelope, assuming that the test results are independent

**Supplementary Figs 2 (a-h)** Regional association plots of loci reaching suggestive significance (*P* < 1 x 10^-6^) in the 23andMe cohort. Blue lines indicate the recombination rate plotted along the right Y-axis. The colors indicate the strength of linkage disequilibrium (LD) with the index SNP. Genes near the lead SNP are shown along the X-axis. The symbol “+” indicates a genotyped SNP; “o” indicates an imputed SNP. **a.** rs192656182 **b.** rs2247036 **c.** rs17044095 **d.** rs137882920 **e**. rs117367662 **f.** rs1650337 **g.** rs75972139 **h.** rs6445791


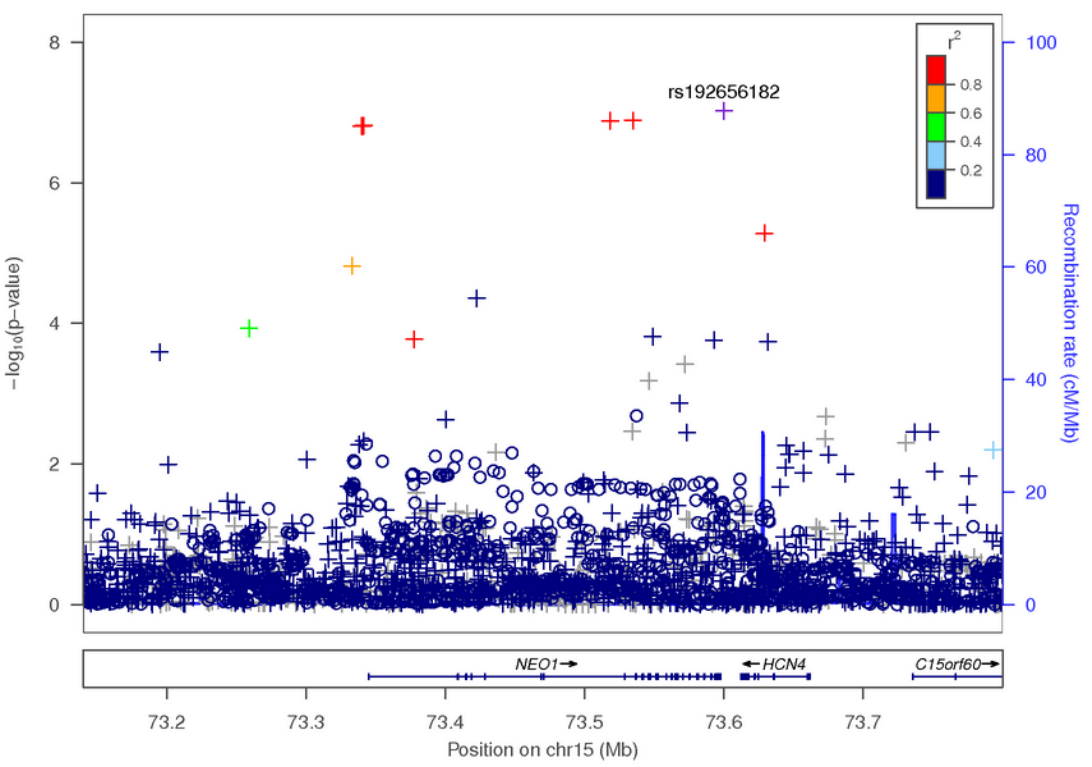


1.
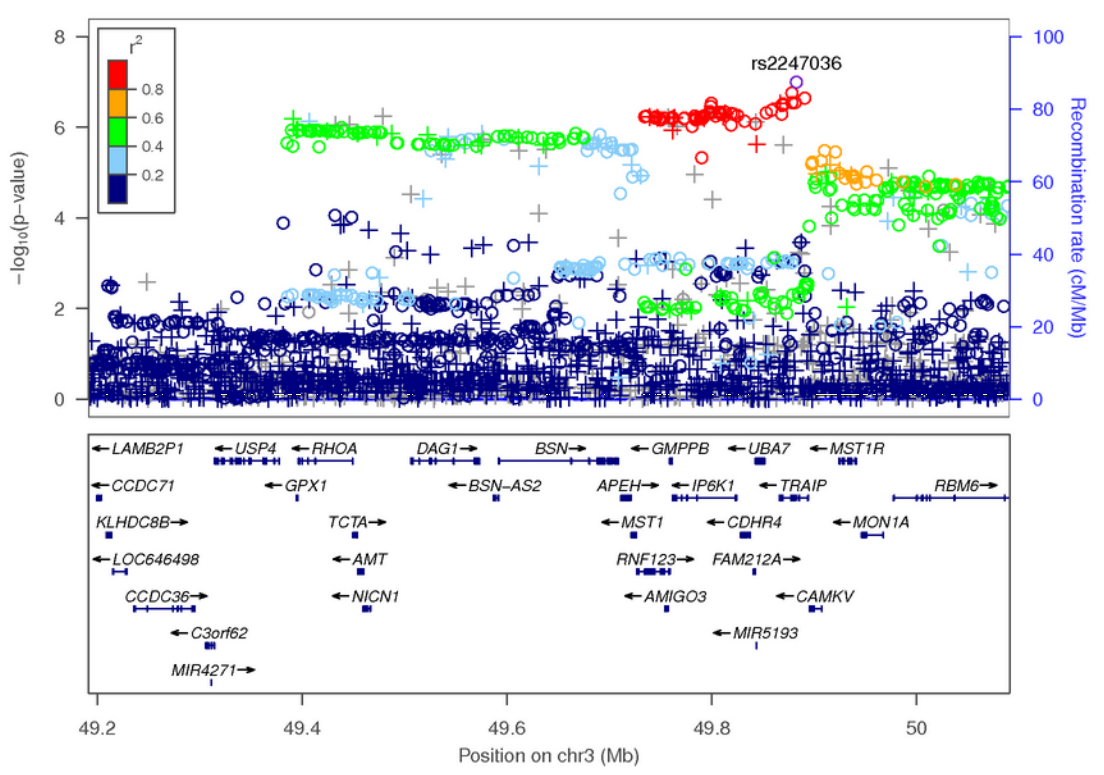


**c.**


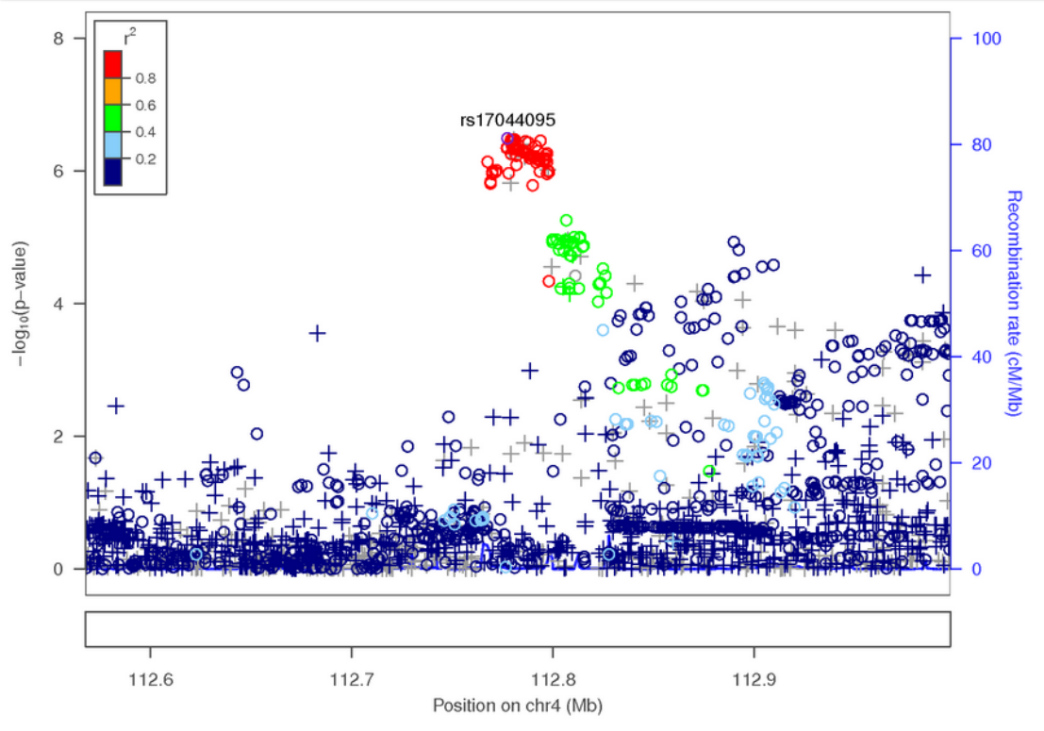


**d.**


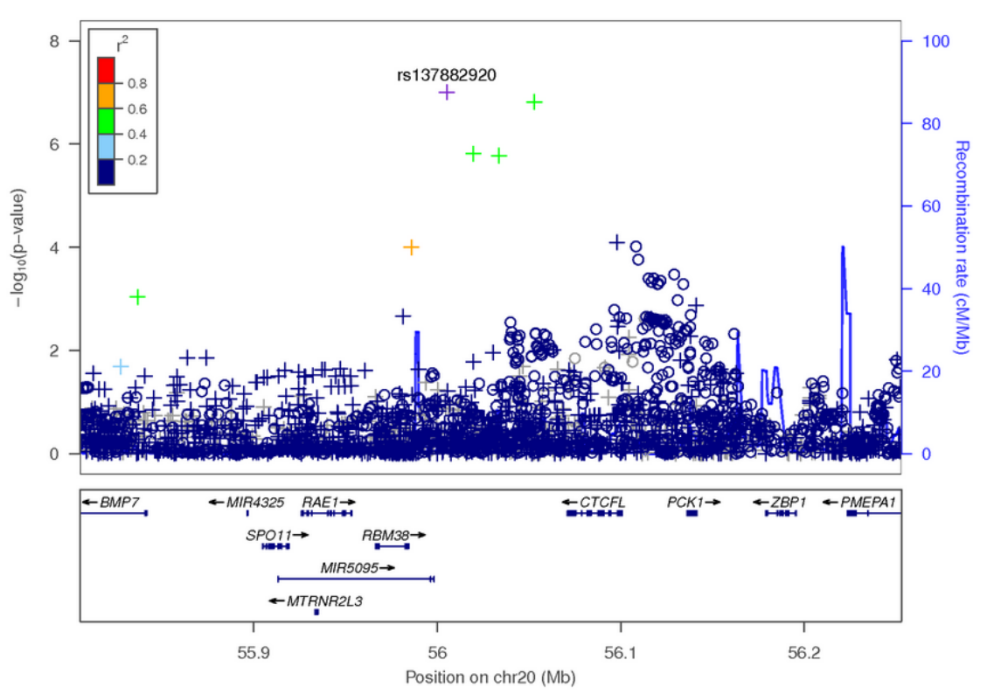


**e.**


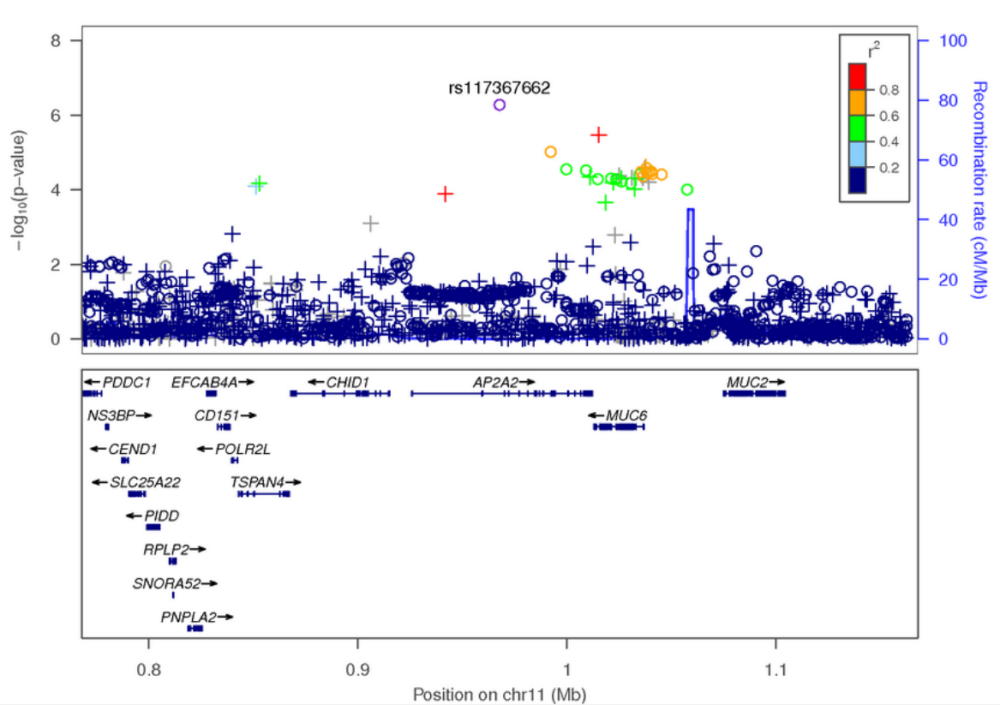


**f.**


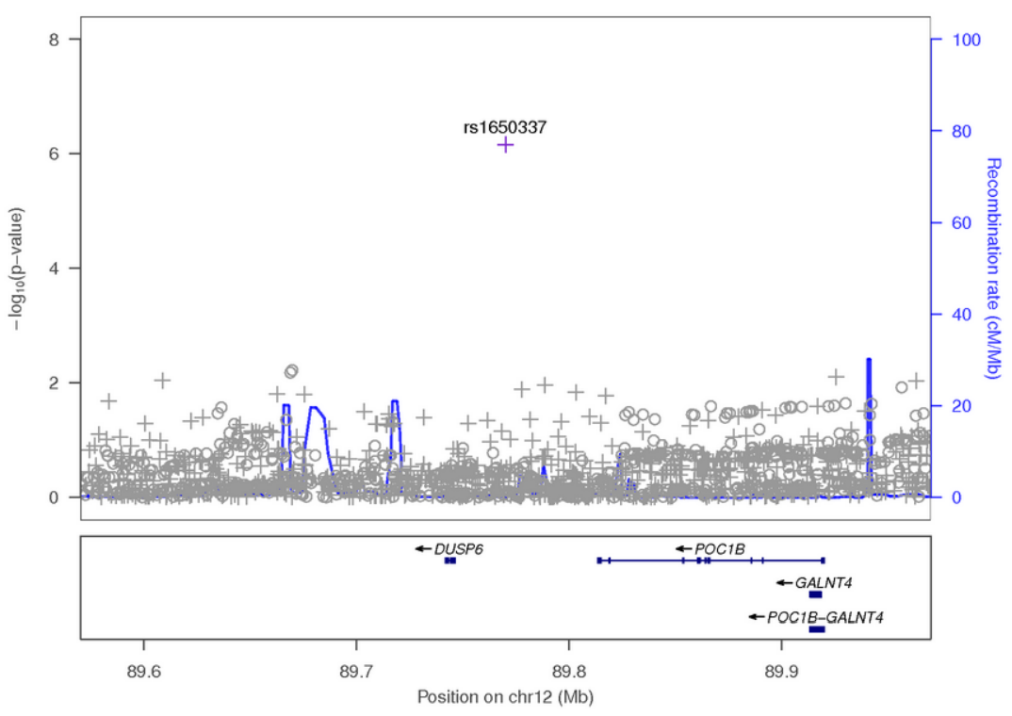


**g.**


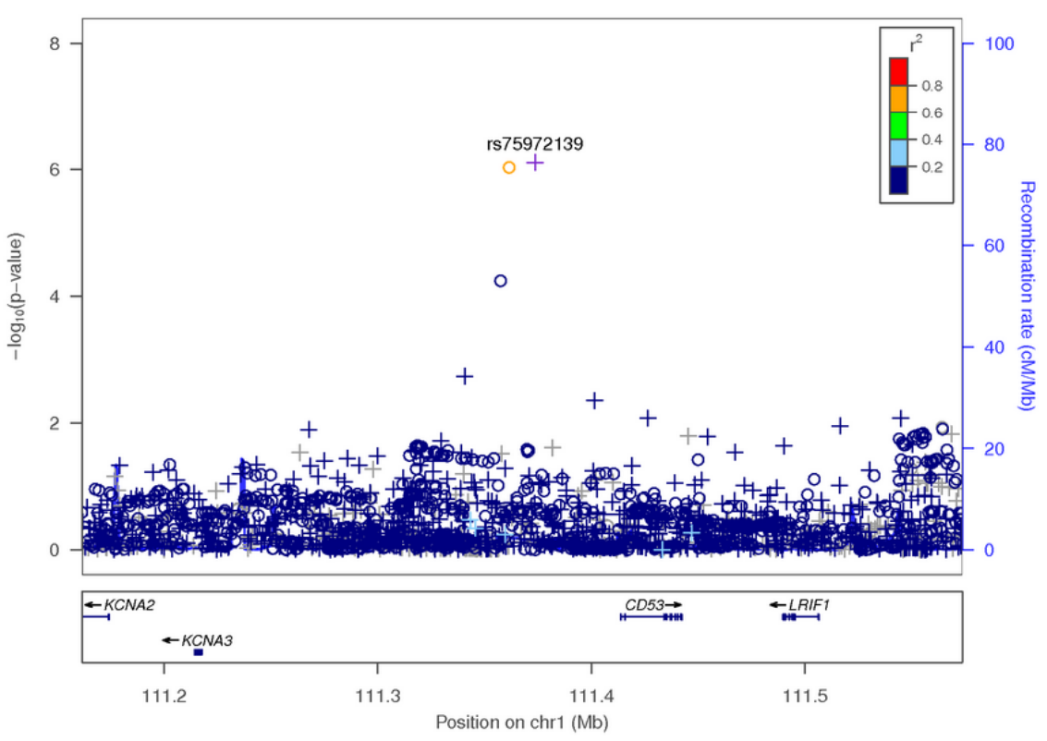


**h.**


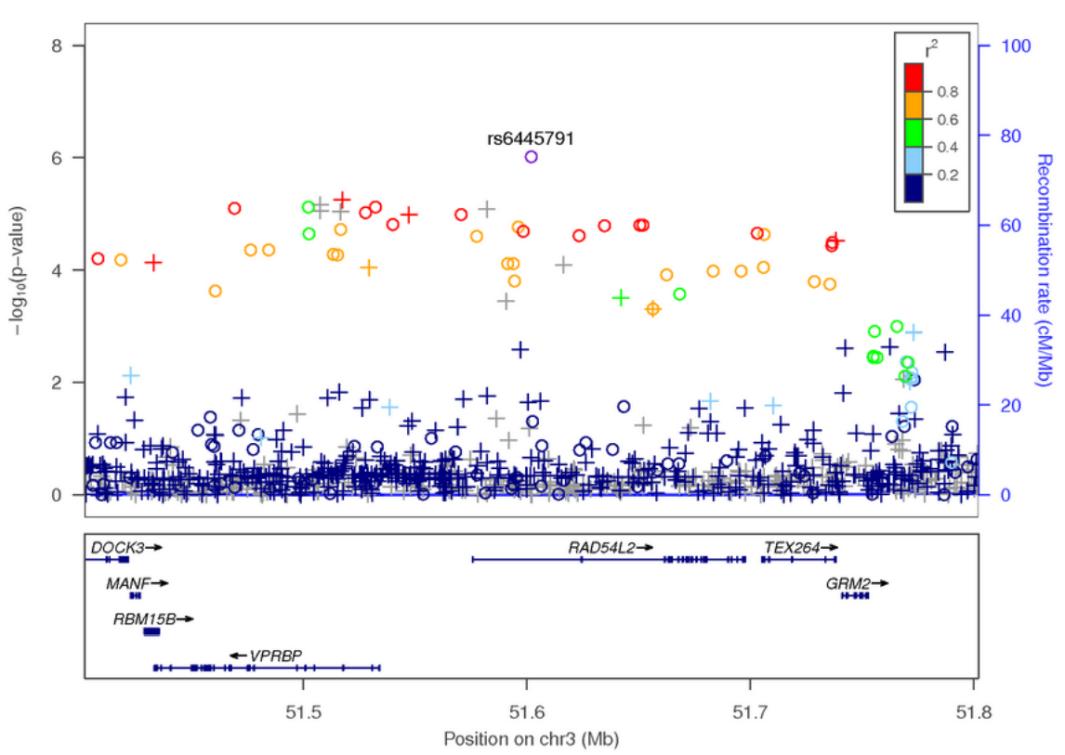


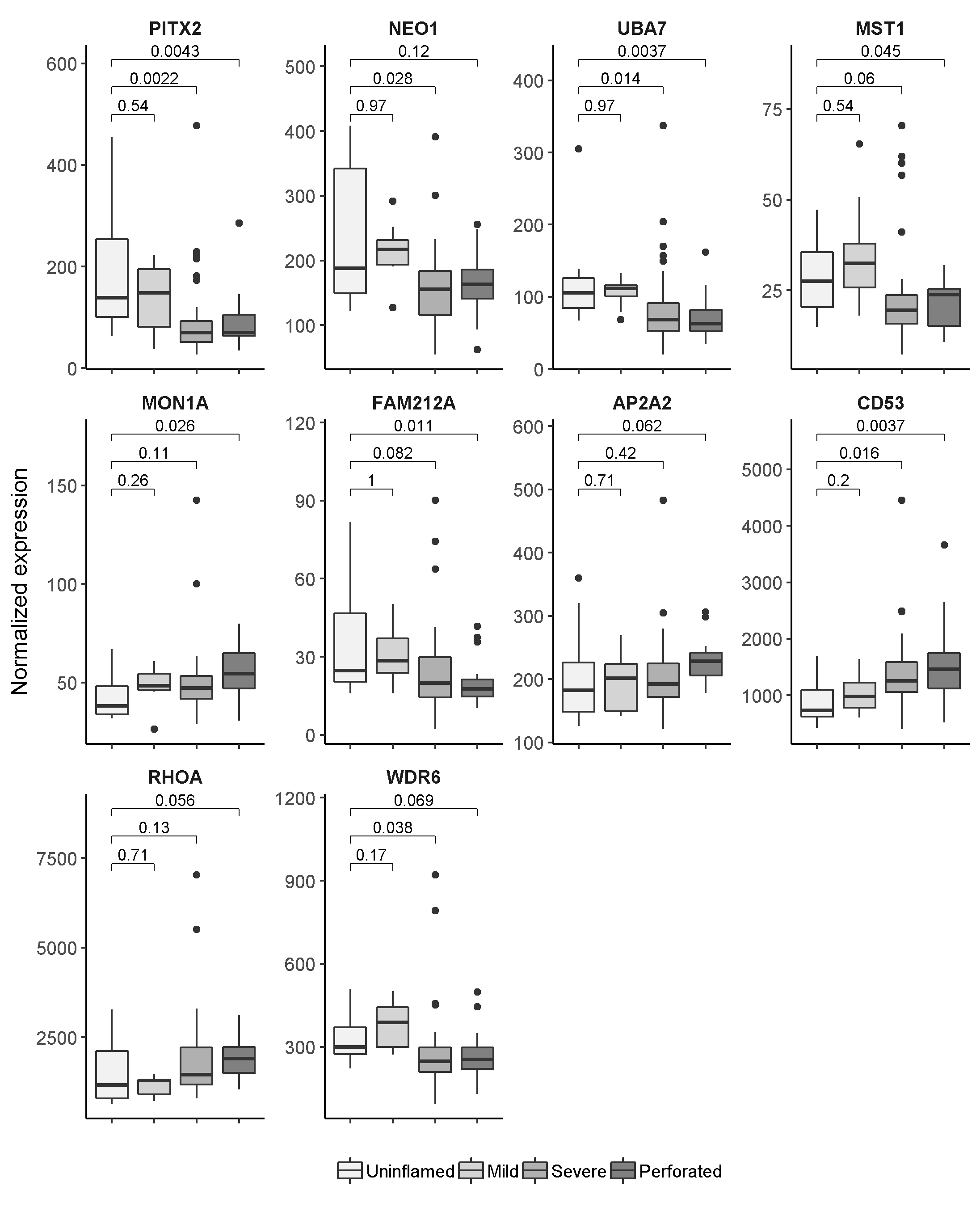


**Supplementary Fig 3** Nominally differentially expressed genes (p-value<0.05) by the Kruskal-Wallis test among uninflamed, mildly inflamed, severely inflamed, and perforated pediatric appendix samples. Pair-wise p-values by Wilcoxon rank sum test are listed. The y axis depicts the normalized count number of transcripts for the ten genes across the classifications of inflammation of resected appendices. Horizontal lines indicate the median expression values, boxes represent the interquartile range, whiskers extend to the most extreme point within 1.5*interquartile range of the box, and points represent extreme values falling outside the whiskers.


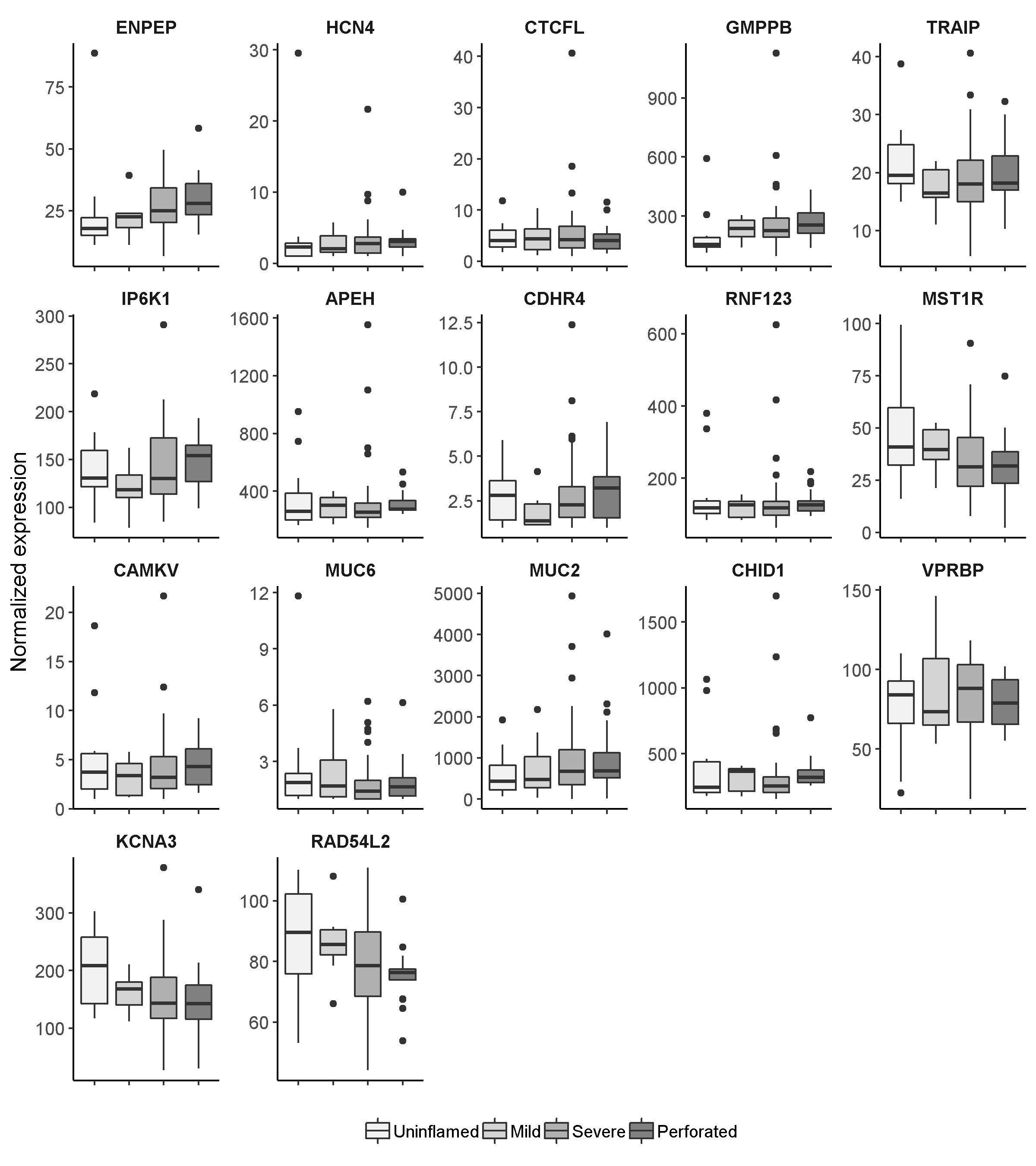


**Supplementary Fig 4** Non-differentially expressed genes by the Kruskal-Wallis test among uninflamed, mildly inflamed, severely inflamed, and perforated pediatric appendix samples. The y axis depicts the normalized count number of transcripts for the ten genes across the classifications of inflammation of resected appendices. Horizontal lines indicate the median expression values, boxes represent the interquartile range, whiskers extend to the most extreme point within 1.5*interquartile range of the box, and points represent extreme values falling outside the whiskers*.*
